# Supplementary material for: GPR171 Agonist Reduces Chronic Neuropathic and Inflammatory Pain in Male, But Not Female Mice
Source: Front Pain Res (Lausanne). 2021 Sep 10;2:695396. doi: 10.3389/fpain.2021.695396 (PMC8915562; doi:10.3389/fpain.2021.695396)
Supplement: Supplementary file 1 [file Data_Sheet_1.docx]

GPR171 Agonist Reduces Chronic Neuropathic and Inflammatory Pain in Male, but not Female Mice

Akila Ram, Taylor Edwards, Ashley McCarty, Leela Afrose, Max V. McDermott, and Erin N. Bobeck

Supplementary Material

##
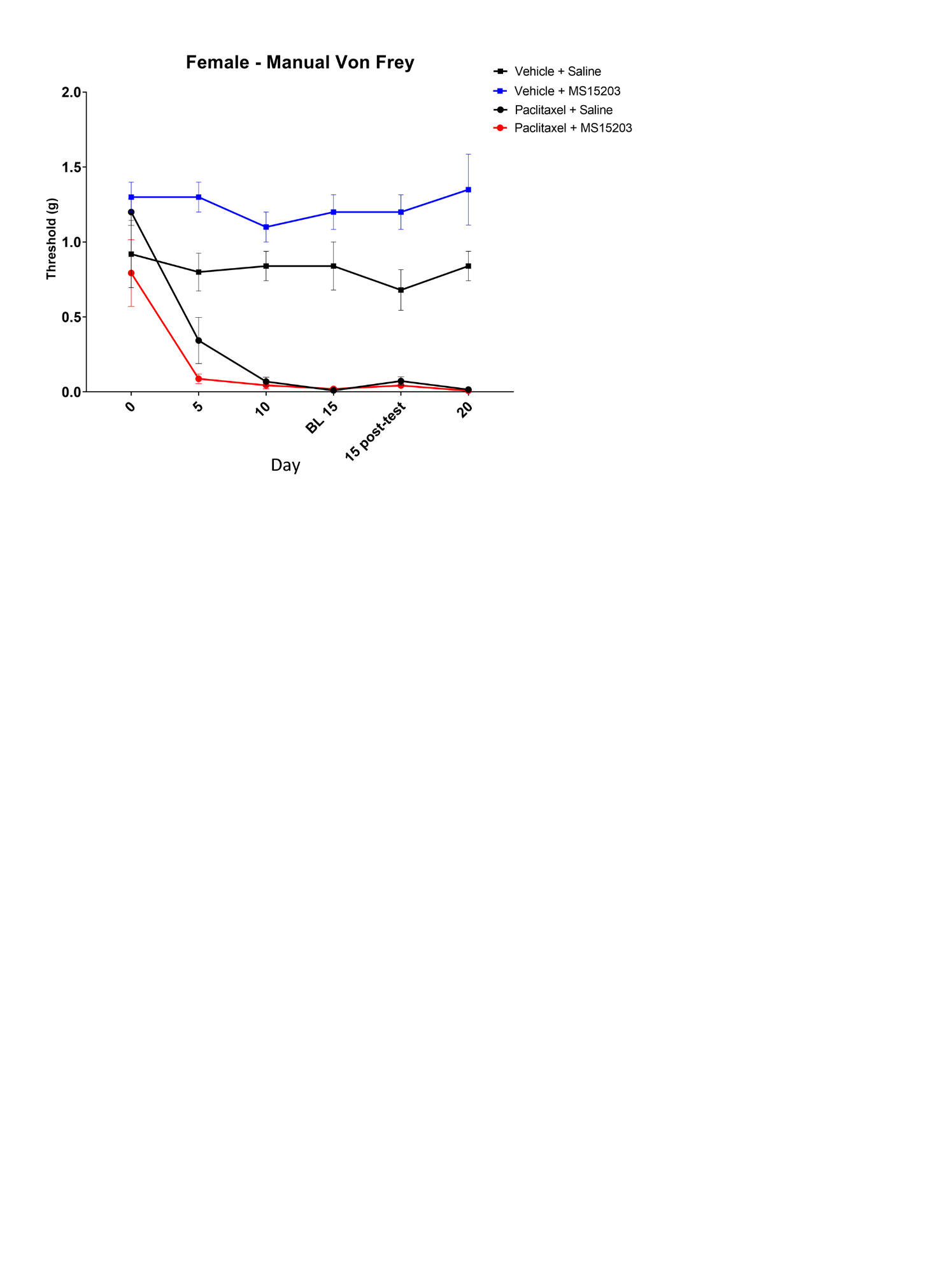


## Supplementary Figure S1. *Neuropathic pain in female C57bl/6CS mice produces allodynia below measurable thresholds of manual von Frey filaments*. Regularly cycling female mice (n=3/group) displayed allodynia arising from paclitaxel-induced peripheral neuropathy and displayed persistent nocifensive responses to applications of the lowest von Frey filament. BL 15 stands for baseline on Day 15 prior to commencement of MS15203 treatment.

##
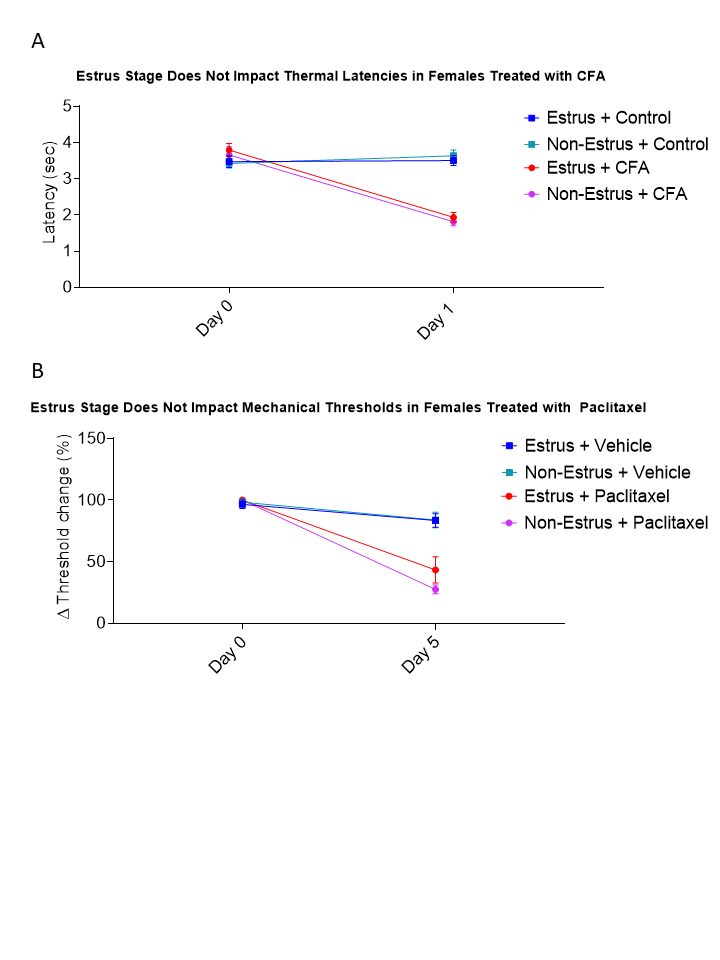


**Supplementary Figure S2.** *Estrus stage does not impact baseline thresholds of female mice.* Female mice do not show significant differences in their A) thermal thresholds or B) mechanical thresholds on Day 0 of the chronic pain studies. Two-way repeated measures ANOVA indicated significant differences in the thresholds of the mice in inflammatory or neuropathic pain. However, a Bonferroni’s post-hoc test revealed that there were no differences in the baseline thresholds of mice in their respective studies.

Ff
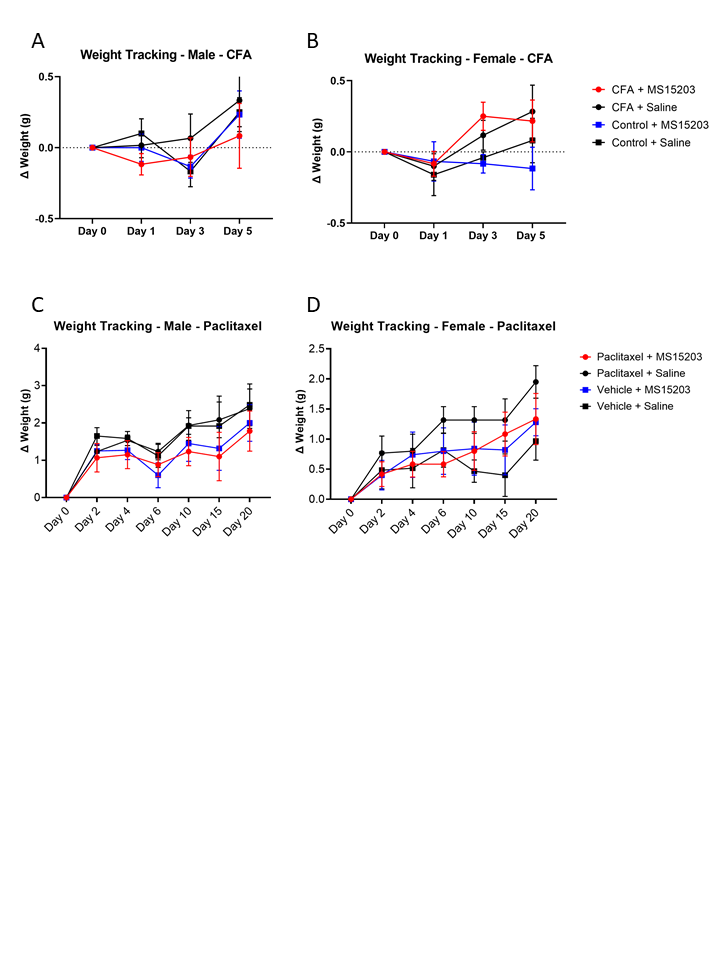


**Supplementary Figure S3.** *MS15203 treatment does not produce significant weight gain in male or female mice.* Once-daily injection of MS15203 (10mg/kg, i.p.) for 5 days does not produce significant weight grain in male (A, C) or female (B, D) mice. A two-way repeated measures ANOVA indicated significant differences in weight changes in mice in the chemotherapy-induced peripheral neuropathy (CIPN) study. However, a Bonferroni’s post-hoc test revealed that there were no significant differences in weight changes between Day 15 and Day 20 of the study, the duration in which the mice received MS15203.


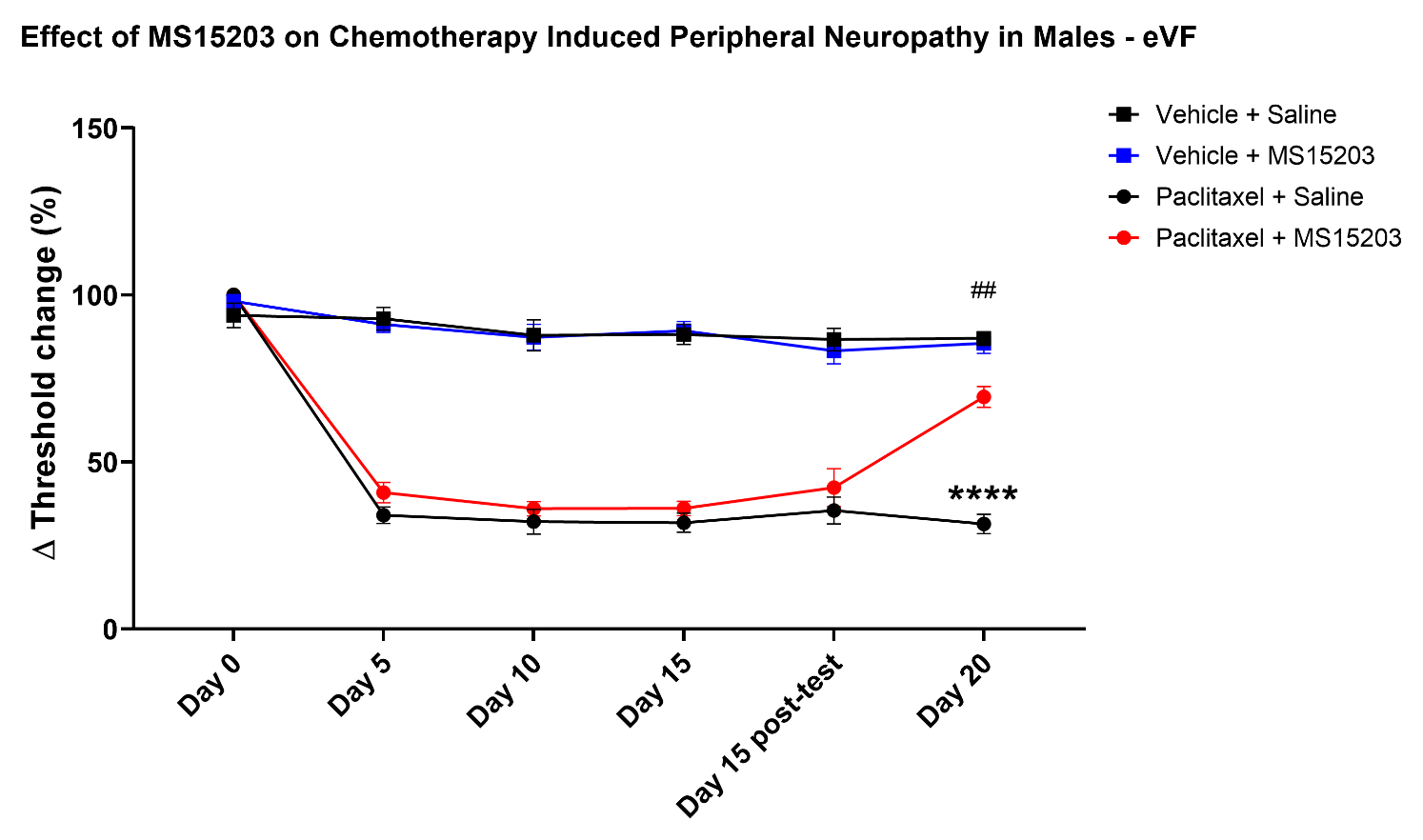


**Supplementary Figure S4**. *MS15203 treatment reduces chronic neuropathic pain in male mice as measured by an electronic von Frey*. Male mice (n=5-6/group) treated with paclitaxel (16 mg/kg cumulative, i.p.) developed allodynia by Day 5 of the study as measured by an electronic von Frey device. Chronic treatment with MS15203 (10 mg/kg, i.p. once daily) from Days 15 through 20 alleviated allodynia by significantly increasing mechanical thresholds compared to Paclitaxel + Saline treated mice. Repeated measures two-way ANOVA with Bonferroni’s post-hoc test. ****p<0.0001 indicates significant differences between Paclitaxel + Saline and Paclitaxel + MS15203 groups. ##p<0.01 indicates significant differences between Paclitaxel + MS15203 and Vehicle + Saline treated groups.

| Name of Gene Target | Forward Primer Sequence | Reverse Primer Sequence |
| --- | --- | --- |
| GAPDH | TGAAGGTCGGTGTGAACG | CAATCTCCACTTTGCCACTG |
| GPR171 | CTGGCGGTGTCTAATTTGTG | TTTTCTTCCAGAGGCTTGCTC |
| PCSK1N | AGTGTATGATGATGGCCC | CCCTAGCAAGTACCTCAG |

**Supplementary Table 1.** *Primer sequences for quantitative RT-PCR.*
